# Core-Shell Morphology of Redispersible Powders in Polymer-Cement Waterproof Mortars

**DOI:** 10.3390/polym10101122

**Published:** 2018-10-10

**Authors:** Stefano Caimi, Elias Timmerer, Michela Banfi, Giuseppe Storti, Massimo Morbidelli

**Affiliations:** Institute for Chemical and Bioengineering, Department of Chemistry and Applied Biosciences, ETH Zurich, 8093 Zurich, Switzerland; stefano.caimi@chem.ethz.ch (S.C.); eliast@student.ethz.ch (E.T.); mbanfi@student.ethz.ch (M.B.); giuseppe.storti@chem.ethz.ch (G.S.)

**Keywords:** core-shell morphology, copolymers, spray-drying, crack-bridging properties

## Abstract

Redispersible powders based on soft core-hard shell polymer particles can be used as additives in polymer-cement mortars. The role of this morphology on the spray-drying production of these powders and on the crack-bridging properties of the corresponding cement-based membranes is investigated. Different polymer latexes at high solid content with varied core-shell ratio, shell thickness and chemical composition (hardness) were prepared from styrene and 2-ethylhexyl acrylate monomers via semi-batch emulsion polymerization. The latexes were characterized in terms of size, composition, and glass transition temperature (*T${}_{g}$*), and spray-dried to obtain redispersible polymer powders (RPPs) using poly (vinyl alcohol) and limestone powder as anti-caking agents. The polymer powders were mixed with a mortar mixture and redispersed in water to produce cement-based membranes, which were tested for crack-bridging properties at different temperatures. The results showed that it was not possible to spray-dry a dispersion of homogeneous polymer particles with *T${}_{g}$* of −25 ${}^{\circ }$C, unless these particles are protected by much harder (high *T${}_{g}$*) shell. In particular, it was observed that a thicker shell improved the spray-ability, but lowered the crack-bridging properties of the produced membrane. A trade-off between these two was revealed to be the key for the optimal design of the polymer nanoparticles, as proven by the systematic study of the core-shell morphology reported in this work. The best compromise was shown to consist of particles larger than 300 nm, shell thickness of about 5 nm, and core-shell ratio of 97%, with styrene content in the shell not larger than 80% to avoid excessive hydrophobicity.

## 1. Introduction

Cement-based materials are the most widely used components in construction industry, thanks to their setting and hardening properties. To improve their tensile strength, water impermeability and chemical resistance, various additives are normally used [1,2,3]. In particular, the addition of polymers is known to improve the workability of fresh mortars, the deformability, the adhesive performance, the cracks and the freezing-thawing resistances of hardened mortars [1,4,5,6,7,8,9,10]. Due to these advantages, polymer-cement mortars (PCMs) are commonly used in a wide variety of applications, including floor screeds, decorative finishing, tile adhesives and waterproofing systems [6]. The latter, in particular, is of large interest for the construction industry, as the PCMs provide a cheap and green alternative to other waterproofing methods, like for example bituminous sheet-based systems, which arrive at the construction site as rolls and are then laid on the surface, thus requiring substantial work as the resulting joints need to be carefully sealed as they are potential weak spots. On the other hand, PCMs can be easily applied using a trowel, roller, spray or a brush and are therefore joint-free. In addition, their thickness can be varied by the user and is not predetermined by the manufacturer, thus widening the possible application range.

PCMs for waterproofing can be divided into two categories, one-component (1C) and two-component systems (2C). The latter comes as a liquid component (containing mainly the latex dispersion) which needs to be mixed with a second dry-mix component (containing sand, cement, and other ingredients) in the right ratio and then to be applied on the supporting structure. These systems, however, are susceptible to human errors during mixing and transport to the construction site is expensive. 1C systems, on the other hand, come as dry-mix mortars (consisting of the sand, cement, redispersible polymer powder (RPPs), and other ingredients), which only needs to be mixed with water on-site. The 1C solution reduces transportation and packing costs and increases the products shelf life [11,12]. A limitation to this approach comes from the redispersibility of the polymer powder, which is not straightforward particularly in the case of relatively soft polymers such as those used for these applications.

To prepare RPPs, techniques like spray- and freeze-drying are commonly used. Both methods exploit the evaporation of water or sublimation of ice under specific conditions of temperature and pressure. Freeze-drying is normally used to produce high-value products, as its operation is relatively complex and costly [13]. Spray drying, on the other hand, is 30–50 times cheaper and can handle various feedstocks like emulsions, slurries and solutions, which makes it the method of choice for drying polymer dispersions to produce RPPs [14,15,16,17].

In a typical spray drying process, the latex dispersion is first atomized through a nozzle and then sprayed into the hot drying chamber where the water is quickly evaporated. Within the drying tower, a constant airflow keeps the particles in the chamber to prevent them from reaching the outlet before being fully dried. When the particles leave the chamber, a cyclone separates the exhaust gas and the dried particles are collected. The outcome of a spray drying process is influenced by several variables, such as its design, the inlet and outlet temperatures, the feed rate, the air flow and the atomization step (i.e., nozzle type and atomizing speed) [18,19]. As the drying process occurs at high temperatures, the polymer particles may coalesce and agglomerate, which obviously lowers their ability to be reversibly dispersed. To prevent this, water-soluble polymers (e.g., poly(vinyl alcohol), PVA) can be added as protective colloids and, in addition, anti-blocking agents, such as silicon dioxide or stearic acid, are normally added to avoid caking during storage [15,20,21,22,23,24,25]. It is worth noting that, as there are increasing concerns about volatile organic compounds (VOCs) in building materials, the production of RPPs from water-based polymer dispersions and the spray drying step guarantee the absence of organic solvents and other organic additives [11,12,26,27].

In this work, a systematic study of the most convenient morphology of the polymer particles used in the RPPs with respect to the spray-ability, film forming characteristics and crack-bridging properties is reported. In these applications, polymers with glass transition temperature ($T_{g}$) lower than −20 ${}^{\circ }$C are used. Since it would not be possible to spray-dry a polymer with such a low $T_{g}$, a harder shell is grown around the soft core to limit particles coalescence and form RPPs. Indeed, core-shell morphologies were intensively studied in the literature and found application in various research areas, including limitation of the particles interpenetration upon drying, controlled release of encapsulated active ingredients, and tuning of the liquid-liquid interfaces [28,29,30,31,32,33]. In particular, for the preparation of RPPs, the core-shell morphology can improve the polymer spray-ability and its redispersion, without affecting the film forming and crack-bridging properties and without further addition of expensive additives [24,34,35,36]. In general, styrene and 2-ethylhexyl acrylate can be taken as hard ($T_{g}$ = 100 ${}^{\circ }$C) and soft ($T_{g}$ = −50 ${}^{\circ }$C) monomers, respectively, while their ratio is selected so to match the desired $T_{g}$. In order to carefully control the copolymer composition and therefore the value of $T_{g}$, semi-batch emulsion polymerization was adopted [37,38] using a reactive emulsifier (surfmer, MAPTAC) as stabilizer.

## 2. Materials and Methods

### 2.1. Materials

Styrene (STY, 99.5% stab. with 10–15 ppm 4-t-butylcatechol, from ABCR, Karlsruhe, Germany) and 2-ethylhexyl acrylate (2-EHA, 98%, from ABCR) were selected as monomers, 2,2${}^{\prime }$-Azobis(2-methylpropionamidine) dihydrochloride as initiator (V-50, 98%, from Acros Organics, Fair Lawn, NJ, USA), and [3-(Methacryloylamino)propyl] trimethylammonium chloride solution (MAPTAC, 50 wt% in H${}_{2}$O, from Sigma Aldrich, Steinheim, Germany) as surfactant or surfmer due to the presence of an unsaturated bond. All materials were used without further purification. Deoxygenated Millipore water (Millipore Synergy, Merck, Darmstadt, Germany) provided the reaction medium for all syntheses. For spray drying fumed silica, dolomite and poly(vinyl alcohol) (PVA, H$\ddot{o}$ppler viscosity 4 mPa·s, hydrolysis degree 88 mol%) were used as received. Chloroform-d (99.8 atom%D, stab. with Ag, from Armar Isotopes, D$\ddot{o}$ttingen, Switzerland) was used as received for NMR characterization. For cement compatibility and crack-bridging tests, Portland cement (CEMI 52.5N Milke classic, from HeidelbergCement, Heidelberg, Germany) and quartz sand (0.1–0.3 mm) were used as provided by the supplier.

### 2.2. Syntheses

All semi-batch emulsion polymerizations were carried out in a 1 L glass-jacketed reactor (Syrris Atlas automated reaction system, Syrris, Royston, UK) fitted with a reflux condenser, sampling device, N${}_{2}$ inlet, two feeding inlets and a PTFE anchor stirrer equipped with two blade impellers rotating at 200 rpm. For the polymerization, the reactor was charged with a solution of the surfactant MAPTAC (IC) in deoxygenized water (bubbled overnight with N${}_{2}$) and heated up to 80 ${}^{\circ }$C using the heating jacket and an oil bath (Polystat CC 302, Huber, Offenburg, Germany). After reaching the reaction temperature (±0.5 ${}^{\circ }$C), part of the initiator solution (IS) was added into the reactor as a shot. The remaining IS and monomer core mixture (CF) were then fed using two pumps (HPLC compact pump, Bischoff, Leonberg, Germany). The initiator was fed for 6 h, while the core monomer mixture was fed for 2.5 to 3.5 h depending on the core-shell ratio used. After switching off the core feed, it was waited for 1 to 1.5 h (WT) until the conversion reached approximately 80%. The monomer shell mixture (SF) was then fed for 0.5 to 1.5 h, in order to have a total monomer feed time of 4 h. After switching off the initiator feed, the reaction was stirred for an additional hour to ensure full conversion. The detailed reaction formulations are summarized in the discussion section and in Tables S1 and S2.

### 2.3. Characterization of Polymer Dispersions

A sample was taken from the polymerization reactor every hour to monitor instantaneous conversion, particle size and composition. By measuring the solid content with a HG53 Halogen Moisture Analyzer (Mettler-Toledo, Columbus, OH, USA) the instantaneous conversion could be calculated. The average radius of the polymer NPs and the polydispersity index (PDI) are measured by dynamic light scattering using Zetasizer Nano ZS 3600 (Malvern Instruments, Malvern, UK) after diluting the sample with deionized water. To analyze the instantaneous composition of the copolymer, the samples were dried in a vacuum oven at 50 ${}^{\circ }$C and dissolved in deuterated chloroform to perform ${}^{1}$H-NMR measurements using a 300 MHz Spectrometer (Bruker, Billerica, MA, USA). By calculating the peak integrals from the spectra it was possible to estimate mole and mass fractions of 2-EHA in each sample. As an example, the spectra for monomers and copolymer are reported in the Supplementary Information in Figure S4 for sample *a*.

The zeta potential of a 0.01 wt% solution of the final latex was measured using the Zetasizer Nano ZS 3600 and the pH was measured using a SevenEasy pH-meter (Mettler-Toledo).

To determine the glass transition temperature ($T_{g}$) of the produced copolymer, differential scanning calorimetry was performed in a Q1000 Differential Scanning Calorimeter (TA Instruments, New Castle, DE, USA) using 10 mg of sample in 40 μL aluminum crucibles, heating and cooling rates of 5 ${}^{\circ }$C min${}^{-1}$ in nitrogen atmosphere, and temperature range from −80 to +100 ${}^{\circ }$C. The $T_{g}$ was obtained from the DSC plot (heat flow vs. temperature) using the inflection point of the S-shape profile, as described in the literature [39]. Finally, to evaluate the film-forming ability, a few droplets of the polymer latex were transferred into a petri dish and dried in air at room temperature.

### 2.4. Spray-Drying

For spray drying in a NiroAtomizer (GEA, D$\ddot{u}$sseldorf, Germany), a water solution of 25 wt% PVA was prepared and added to the copolymer dispersion to have 15% of PVA with respect to the polymer. Then, the dispersion was diluted with water to have a total solid content of 25%. The inlet temperature of the spray-drier was set at 135 ${}^{\circ }$C and the outlet temperature was kept at approximately 70 ${}^{\circ }$C. A peristaltic pump (ISM 817, IKA, Staufen im Breisgau, Germany) was used to feed the dispersion at 12.2 g min${}^{-1}$. To prevent caking of the dried powder, silica was fed together with limestone powder with a ratio of 1:18 through a dry powder feeder (AccuRate, Schenck Process Europe, Darmstadt, Germany) with a feed rate of 0.75 g min${}^{-1}$ to have 19 wt% with respect to the polymer. The compressed air inlet to disperse the anti-caking mixture was set at 2 bar and the spray nozzle was set at 3.5 bar. The amount of collected powder was weighted to measure the yield and 0.5 g of it was mixed with 10 mL of water to test redispersibility.

### 2.5. Polymer-Cement Mortars

To test the quality of the cement-based mortars prepared using the different copolymers, 25 g of dried polymer were dry-mixed with 56 g of quartz sand and 19 g of Portland cement. The dry-mix was poured into approximately 20 g of water and a timer was started. It was then stirred vigorously for 1 min and the wetting speed as well as the fluidity was analyzed. If the amount of water was not sufficient to yield a creamy consistency, additional water was used. The polymer-cement mixture was then applied on top of two adjacent cement fiber plates with a thickness of 2 mm. 5 h later, a second layer of 2 mm polymer-cement membrane was applied. The membrane was left at rest for 28 days at standard conditions (23 ${}^{\circ }$C/50% relative humidity) before the crack-bridging test.

The mortar and sample preparation procedure is sketched in Figure S2 in the Supporting Information.

### 2.6. Crack-Bridging Ability of the Polymer-Cement Mortar

Crack-bridging tests were performed according to EN 14891 [40] in the temperature range from −20 to +23 ${}^{\circ }$C using a Z020/TH2S (Zwick/Roell, Ulm, Germany) to measure the maximum expansion until visible cracks form. The displacement rate is 0.15 mm min${}^{-1}$ and is kept constant during the test. Before the test and the recording starts, a pre-load of 20N was applied. To be able to vary the testing temperature, the whole straining device was encased by a temperature-controlling unit (TEE 65/40X, RS-Simulatoren).

## 3. Results and Discussion

### 3.1. Synthesis of Core-Shell Polymer Nanoparticles

**Figure 1:**
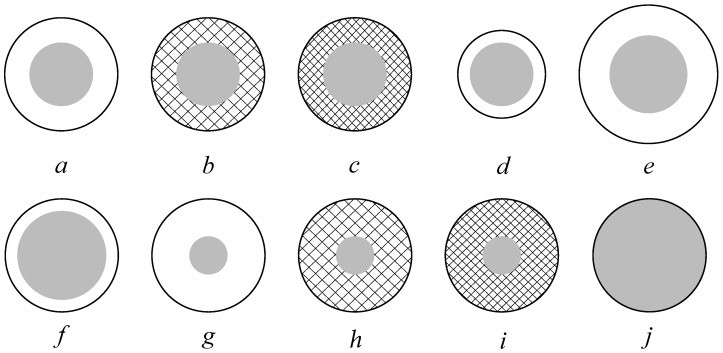
Morphologies of the different synthesized core-shell polymer nanoparticles. Medium core size with medium shell thickness and styrene content of (**a**) 80%, (**b**) 90%, and (**c**) 99% in the shell. Medium core size with a (**d**) thinner and (**e**) thicker shell of styrene content of 80% and different overall particle size. Different core size while keeping the overall size constant with respect to (**a**), leading to a (**f**) thinner and (**g**) thicker shell of styrene content of 80%. Same morphology as (**g**) but with a styrene content of (**h**) 90% and (**i**) 99% in the shell. (**j**) Core-only latex particles.

Core-shell polymer nanoparticles with the different morphologies sketched in Figure 1 were prepared in order to investigate the effect of core-shell ratio, shell thickness, and shell hardness on the ability to be spray dried and to form crack-resistant membranes. The core is made of 25/75 wt% of STY/2-EHA, respectively, having a $T_{g}$ of approximately −25 ${}^{\circ }$C and with a size ranging from 255 to 285 nm, as reported in Table 1. Being the core nanoparticles extremely soft (i.e., very low $T_{g}$), a dispersion of this product could not be spray dried as it led to fully coalesced particles which could not be redispersed. To improve the spray-ability, a harder shell with $T_{g}$ higher than 65 ${}^{\circ }$C was grown around the soft core.

The different types of particle morphologies described in Table 1 were prepared. On one hand, the shell thickness was varied from 5 to 15 nm while keeping the core size constant at approximately 280 nm (latexes *a*, *d* and *e*). On the other hand, the final particle size was kept constant at approximately 300 nm and the core-shell ratio was varied from 87 to 97% (latexes *a*, *f* and *g*). Moreover, to study the influence of the hardness of the shell, its composition was varied from 80 to 99% STY content, while keeping the core size and shell thickness constant at 280 and 300 nm (latexes *a*, *b* and *c*) and at 260 and 295 nm (latexes *g*, *h* and *i*), respectively. Finally, for comparison purposes, a latex made of core-only particles (i.e., with an overall composition of 25/75 wt% of STY/2-EHA) was considered (latex *j*).

**Figure 2:**
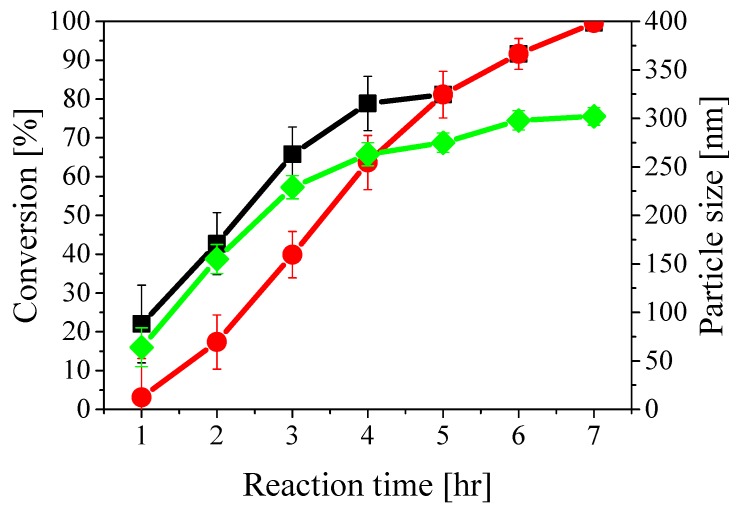
Instantaneous (black squares) and cumulative (red circles) conversion and average particle size (green diamonds) during the synthesis of sample *a*.

The values of final particle size and PDI (polydispersity index by DLS) for all particles are reported in Table 1. Small PDI values (below 0.1) indicate narrow particle size distribution, meaning that almost all particles have the same size. As above-mentioned, particle size, instantaneous conversion and copolymer composition were monitored during all reactions. With illustrative purposes, the time evolutions of instantaneous and cumulative conversion and average particle size in the case of reaction *a* are shown in Figure 2. These curves had similar shape for all other reactions and, in particular, it is possible to note that both particle size and conversion values show a rapid increase at the beginning, while slowing down after about 4 h of reaction time due to the progressive monomer depletion. The initial lag time before the reaction starts is due to the presence of inhibitors in the monomers along with the lower reactivity of 2-EHA with respect to STY. On the other hand, the instantaneous conversion (defined with respect to the amount of monomers added to the reactor until the given time) reaches a value of approximately 70% after 3 h and remains larger for the rest of the reaction. The cumulative conversion (defined with respect to the entire amount of monomers added into the reactor) grows following the typical S-shape and reaches 100% after 7 h. The two curves obviously superimpose after 5 h when the monomer mixture addition was completed. The average particle size increases up to 300 nm (green curve in Figure 2) and the PDI value was measured to be below 0.1 throughout the whole reaction. The total number of polymer particles remained constant at approximately 1.5 × 10${}^{16}$, indicating no secondary nucleation as reported in Figure S1 in the Supplementary Information.

**Figure 3:**
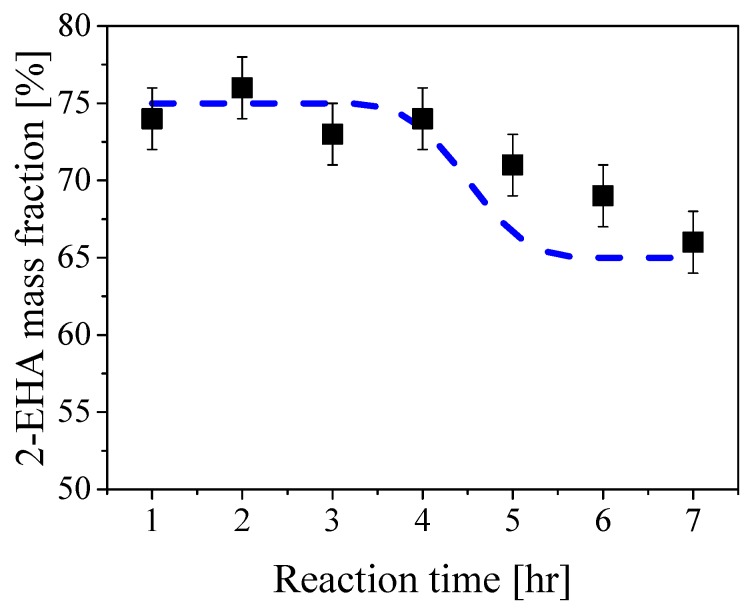
Mass fraction of 2-EHA in the polymer particles for sample *a* during the reaction. The dashed curve represents the cumulative polymer composition corresponding to the fed monomer mixture assuming complete conversion.

The values of the cumulative composition of sample *a* during the reaction are shown in Figure 3. As reported in Table S1, the core mixture (CF) made of STY/2-EHA 25/75 wt% was fed during the first 3 h of the reaction, followed by 1 h rest (WT) and subsequent feed of the shell mixture (SF) (STY/2-EHA 80/20 wt%) for 1 h. This feed policy is consistent with the composition profile in the figure, which is approximately equal to 75% 2-EHA for the first 4 h and then decreases progressively to the value of 65% 2-EHA, which is the expected final cumulative composition assuming complete conversion (dashed curve in Figure 3). These results show no preferential incorporation of the two monomers, with the formed polymer chains having the desired cumulative composition. This is confirmed for all polymer samples as reported in the Supplementary Information, Table S4.

**Figure 4:**
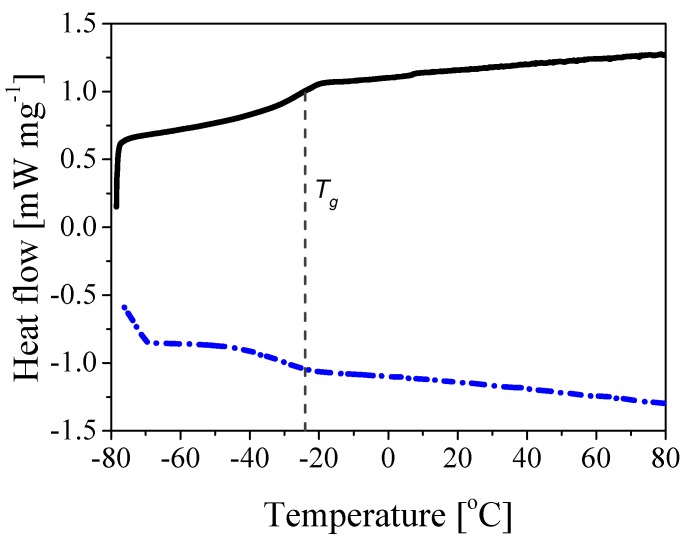
DSC curves of polymer *a*: heating (solid black) and cooling (dashed blue) heat flow profiles.

The $T_{g}$ values of the different copolymers measured by DSC are also shown in Table 1. Approximately −25 ${}^{\circ }$C was estimated in all cases, which is in agreement with the estimated value for a copolymer with composition STY/2-EHA 25/75 wt% using the Fox equation [41,42]:

(1) $$\frac{1}{T_{g}}=\frac{\omega_{1}}{T_{g,1}}+\frac{\omega_{2}}{T_{g,2}}+...+\frac{\omega_{n}}{T_{g,n}}$$

where $\omega_{k}$ and $T_{g,k}$ are the weight ratio and the glass transition temperature of the generic monomer *k*, respectively. This property needs to be closely controlled in the application considered in this work since the polymer $T_{g}$ is known to affect film formation, film shape and cement hydration in mortars [43]. Note that the reported $T_{g}$ values refer to the core only, as it was not possible to observe the characteristic S-shape at higher temperatures corresponding to the shell composition, because the core-shell transition is quite smooth and, therefore, there is a very gradual change in the composition towards the harder shell. This is illustrated, for the case of polymer *a*, in Figure 4 where the heating and cooling profiles are shown. It is seen that only the $T_{g}$ value of the particle core can be estimated from the S-shaped profile of the heating curve (black curve). The shell $T_{g}$ values of the different samples reported in Table 1 were measured by synthesizing particles with homogeneous morphology and the same composition as the shell, that is STY/2-EHA equal to 80/20, 90/10 and 99/1 wt%, which resulted to be 65.8, 74.2, 86.7 ${}^{\circ }$C, respectively.

Finally, to quantify the surface charge of the polymer particles, which allows inferring the stability of the corresponding polymer latexes, the ζ potential values were measured. As the surfactant used in the polymerization bears a positive charge at neutral pH, all measured values of ζ potential are positive and lie between 47.4 and 54.5 mV (Table S3 in the Supporting Information). These electrostatic repulsion forces result in stable dispersion along with good spray-ability during the drying step. Moreover, all latexes were dried into petri dishes to verify their film formation ability. In all cases, the presence of the harder shell does not hinder the film formation upon drying, as reported in Table S3 in the Supporting Information. The used surfmer MAPTAC combines in its molecular structure a charged, positive group, which gives stability to the system, and a polymerizable double bond, which can react with the monomers and be incorporated into the growing polymer chains [44]. Being covalently attached to the particles, it will not desorb during film formation and therefore the properties of the RPPs remain unchanged [45,46,47]. Indeed, conventional surfactant due to their possibility of migrating from the particle surface to the liquid solution are known to negatively affect the final properties of the materials, such as the adhesion strength, the water resistance and the crack-bridging properties [48,49].

### 3.2. Spray Drying and Redispersibility

All the latexes were spray dried according to the procedure described in the experimental section. The values of the size of the obtained powder grains estimated by visual inspection are reported in Table 2 along with their free-flowing ability after 24 h of storage (i.e., no caking effect) and redispersibility in water.

**Figure 5:**
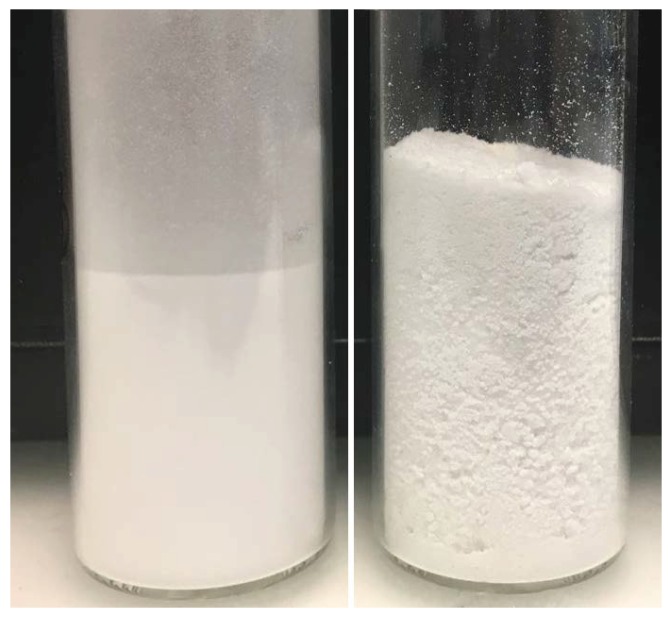
Sample *i* (**left**) and sample *d* (**right**) after spray drying.

Polymer *j*, whose particles are made of soft core only, with a $T_{g}$ of approximately −25 ${}^{\circ }$C, could not be spray dried: the powder stuck to the internal wall of the tower and no dried product could be collected at the unit outlet. However all latexes with core-shell polymer particles could be effectively spray dried. In particular, the samples with low core-shell ratio and thick shell (i.e., latexes *e*, *g*, *h* and *i*) showed the best performance during spray drying. Their hard shell prevented particle interpenetration after aggregation and, therefore, free-flowing, fine powders were produced. To appreciate the difference between coarse and fine powders, a picture of both—coarse powder (polymer *d*) and fine powder (polymer *i*)—is shown in Figure 5. Latex *d* was the only exception, since a non-free-flowing coarse powder was collected in this case. This fact can be understood by considering that this latex has the thinnest shell, probably too thin to exclude interpenetration, and the smallest particle size, both triggering film formation. On the other hand, latex *f*, having the same shell thickness as latex *d*, showed good spray-ability. This may be due to the larger overall particle size, which facilitates the spray drying: since the polymer particles exhibit lower specific surface, they are less exposed to interpenetration at constant amount of protective colloid.

From the data in Table 2, it is also evident that the increasing hardness of the polymer surface going from latexes *a* to *b* and *c* and from *g* to *h* and *i* did not show any visible effect on the powder quality, thus confirming that particle size and shell thickness are the most important factors limiting interpenetration. It can then be concluded that the core-shell structure improves the spray-ability while retaining film-forming properties, as confirmed by the results summarized in Table 2 and Table S3.

About the operating conditions of the spray dryer, it was observed that too low inlet temperatures (lower than 120 ${}^{\circ }$C) resulted in insufficient drying and high residual humidity, leading to the caking effect after 24 h of storage. On the other hand, at too high inlet temperatures (higher than 150 ${}^{\circ }$C), the polymer was too soft and the particles excessively interpenetrated, leading to too coarse powders. Concerning the amount of PVA (15 wt%) and anti-caking agent (19 wt%), it was observed that lower values led to coarser powders as the protective effect against interpenetration was reduced. Higher values were not investigated as they could negatively affect the crack-bridging performance of the final membrane.

**Figure 6:**
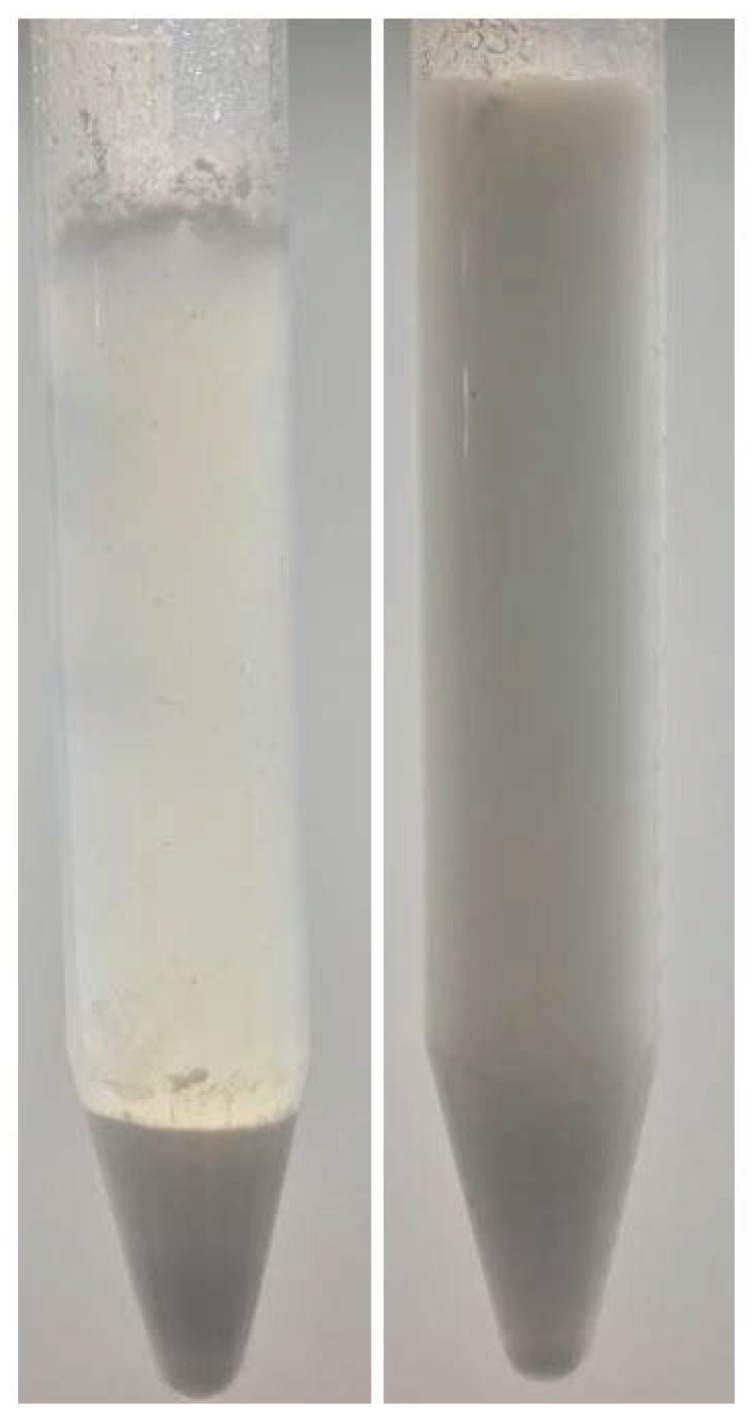
Powders *i* (left) and *d* (**right**) redispersed after spray drying.

As anticipated, to be properly mixed with cement and form homogeneous membranes, the spray dried polymer powders have to be well redispersible in water. Redispersibility was then analyzed as described above and the results are summarized in Table 2 for the different polymer powders. All samples could be easily redispersed by simple stirring and none of them formed large coagulates. However, some powders settled after resting for 24 h, whereas others remained uniformly dispersed. The former are listed as bad redispersible (*br*) and the latter as good redispersible (*gr*) powders in Table 2. The results show that good spray-ability does not necessarily mean that the dried powders are well redispersible. For example, in the case of the samples shown in Figure 5, the coarser powder *d* is better redispersible than the finer *i*, as shown in Figure 6. This can be understood by taking into account the hydrophobicity of the shell: since harder shells have higher styrene contents, their more hydrophobic nature leads to more difficult wetting, which makes them difficult to be redispersed in water. This becomes evident when comparing the redispersibility of powders *a* with *b* and *c* and of the powders *g* with *h* and *i* (see Table 2).

### 3.3. Crack-Bridging and Polymer-Cement Compatibility

**Figure 7:**
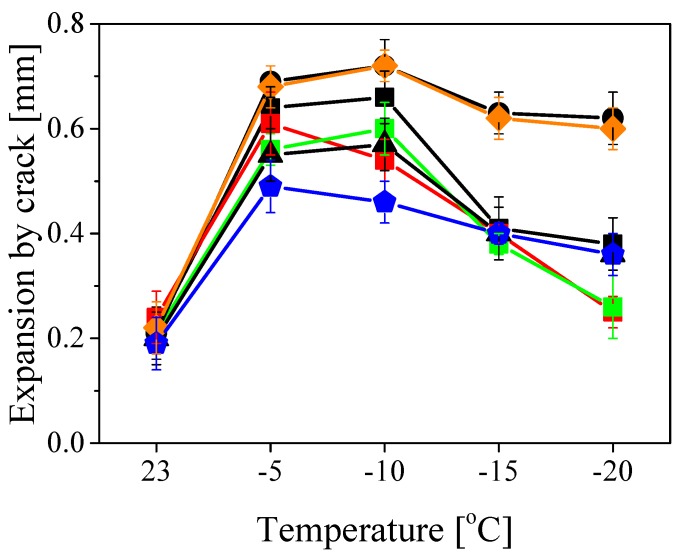
The expansion in millimeters before the membrane cracks at different temperatures for the membranes obtained with the samples *a* (black squares), *b* (red squares), *c* (green squares), *d* (black circles), *e* (black triangles), *f* (orange diamonds) and *g* (blue pentagons).

The polymer powders *a* to *i* were used to produce 2 mm cement-based membranes by mixing the powders with quartz sand, cement and water as described in the experimental section. The membranes were tested for crack-bridging properties and the results are shown in Figure 7. Samples *h* and *i* could not be tested as they did not form homogeneous, crack-free membranes (see Figure S3 in the Supporting Information). This could be imputed to the strongly hydrophobic nature of the shell of these particles which contains high amounts of STY.

The crack-bridging results showed a similar trend for all tested samples (polymers *a* to *g*). At temperatures above 20 ${}^{\circ }$C the performance was poor, as the polymer is excessively soft and unable to bridge cracks in the composite material. As the temperature decreases, clear differences among the samples emerge. In the whole temperature range, samples *d* and *f* (those with thinner shell) showed the largest possible expansion of approximately 0.7 and 0.6 mm at −10 and −20 ${}^{\circ }$C, respectively. In comparison, polymers *e* and *g* (those with thicker shell) showed the lowest performance, reaching about 0.6 (*e*) and 0.5 mm (*g*) at −10 ${}^{\circ }$C and 0.4 mm at −20 ${}^{\circ }$C. This can be understood by taking into consideration that the polymer particles have to fully coalesce during drying in order to avoid cracks formation. A thicker shell does not impede film formation (cf. Table S3) but it increases the fraction of film with higher $T_{g}$, which does not contribute to improve the crack-bridging behavior.

Furthermore, when comparing the samples with medium shell thickness and different styrene content in the shell (polymers *a* to *c*), it is possible to see that harder shell leads to lower performance. Indeed, the membranes prepared using samples *b* and *c* (with 90% and 99% styrene in the shell, respectively) could expand less before cracks form with respect to sample *a*, with 80% styrene in the shell. Again, harder shell makes it more difficult for the polymer particles to fully coalesce and, therefore, to form a crack-resistant film. In general, the performance of all samples decreased at low temperatures, since the polymer gets harder and more brittle as the $T_{g}$ of the polymer (–25 ${}^{\circ }$C) is approached.

## 4. Conclusions

PCMs are widely used in the construction industry for their attractive application characteristics, particularly with respect to waterproofing. Core-shell polymer particles can be used to specifically improve the spray-ability and crack-bridging properties of the membrane obtained from these powders. In order to better understand these systems and eventually optimize the core-shell morphology, various low $T_{g}$ polymer latexes made of styrene and 2-ethylhexyl acrylate were synthesized via semi-batch emulsion polymerization. In particular, the core-shell ratio and the shell thickness and hardness were varied systematically to analyze the system behavior. Tuning the composition of the copolymer, the core had a $T_{g}$ of −25 ${}^{\circ }$C, whereas the shell, richer in styrene, higher than 65 ${}^{\circ }$C. The latexes were spray dried after the addition of 15% of poly (vinyl alcohol) by weight with respect to the polymer as a protective colloid and of a mixture 1 wt% of silica and 18 wt% of limestone powder as anti-caking agent. All obtained powders were redispersed in water and mixed with cement and quartz sand to form mortars which were tested for crack-bridging properties at different temperatures.

It was observed that all samples with core-shell morphology could be effectively spray-dried and led to redispersible polymer powders. In particular, for the system under consideration, the latexes with a particle size larger than 300 nm and a shell thicker than 10 nm exhibited the best performance during spray-drying. Smaller particles and thinner shells led to more interpenetrated particles during drying and therefore formed coarser powders. Moreover, samples containing more that 80% of styrene in the shell resulted in non-uniform and non-crack-free membranes, most probably because of their excessive hydrophobic character. On the other hand, concerning the crack-bridging properties, the mortars formed with the polymers having 5 nm-thick shell with 80% styrene showed best performance, as the particles could fully interpenetrate and coalesce. Therefore, the best compromise in terms of core-shell morphology for spray-ability and highest crack-bridging properties of the final mortar consists of large particles with thin shell (core-shell ratio of 97%, i.e., shell thickness of a few nanometers) and styrene contents in the shell not larger than 80%, that is with limited hydrophobicity.

In conclusion, this study shows the importance of core-shell morphology in improving the performance of PCMs, but also indicates the need for its careful design in terms of geometrical and chemical characteristics.

## Figures and Tables

**Table 1:** Final size, PDI (polydispersity index) and glass transition temperature ($T_{g}$) of the different polymer particles.

Sample	*a*	*b*	*c*	*d*	*e*	*f*	*g*	*h*	*i*	*j*
Core size [nm]	280	278	284	276	279	285	255	258	260	296
NPs size [nm]	302	296	301	286	311	294	293	294	303	
PDI	0.01	0.03	0.02	0.06	0.04	0.02	0.07	0.06	0.01	0.02
$T_{g}$ Core [${}^{\circ }$C]	−24.0	−27.6	−24.2	−25.6	−25.0	−24.4	−21.5	−25.2	−22.7	−24.7
$T_{g}$ Shell [${}^{\circ }$C]	65.8	74.2	86.7	65.8	65.8	65.8	65.8	74.2	86.7	

**Table 2:** Results of the visual analysis of the spray dried polymer latexes. Qualitative grain sizes range from fine (fp) over medium (mp) to coarse (cp) powder. The samples are also classified in free-flowing (ff) and caking (ca) powders. Their redispersibility in water ranges from good (gr), to medium (mr) and to bad (br).

Sample	*a*	*b*	*c*	*d*	*e*	*f*	*g*	*h*	*i*
Grain size	mp	mp	mp	cp	fp	mp	fp	fp	fp
Free-flowing properties	ff	ff	ff	ca	ff	ff	ff	ff	ff
Redispersibility	gr	mr	mr	mr	gr	gr	mr	br	br

## Supplementary Materials

The following are available online at http://www.mdpi.com/2073-4360/10/10/1122/s1, Figure S1: Number of polymer particles in the reactor during the synthesis of sample *a*, Figure S2: Sketch of the membrane preparation procedure for crack-bridging tests, Figure S3: Cement-based membrane obtained with polymer *h* and *i*, after spray drying, Figure S4: NMR spectra for the monomers STY and 2-EHA and for the copolymer STY/2-EHA *a*, Table S1: Reaction formulations for different core-shell particles for samples *a–e*, Table S2:, Reaction formulations for different core-shell particles for samples *f–j*, Table S3: Surface zeta potential, pH value and film-forming abilities of synthesized latexes, Table S4: 2-EHA content of the cumulative polymer particles during the reaction.
