# Supplementary material for: Core-Shell Morphology of Redispersible Powders in Polymer-Cement Waterproof Mortars
Source: Polymers (Basel). 2018 Oct 10;10(10):1122. doi: 10.3390/polym10101122 (PMC6403678; doi:10.3390/polym10101122)
Supplement: Supplementary file 1 [file polymers-10-01122-s001.pdf]

Supplementary Materials: Core-Shell Morphology of Redispersible Powders in Polymer-Cement Waterproof Mortars

Stefano Caimi, Elias Timmerer, Michela Banfi, Giuseppe Storti and Massimo Morbidelli *

1. Syntheses conditions

The exact amounts and conditions of the synthesis of the samples are shown in Tables S1 and S2.

Table S1. Reaction formulations for different core-shell particles for samples *a-e*. All reactions were run at 80 °C with a stirring speed of 200 rpm.

	Sample	*a*	*b*	*c*	*d*	*e*
IC	MAPTAC (50 wt.%) [g]	15.1	15.1	15.1	13.6	16.6
	Water [g]	370	370	370	370	370
IS	V-50 [g]	0.9	0.9	0.9	1	1
	Water [g]	10	10	10	11	11
CF	STY [g]	42	42	42	42	42
	2-EHA [g]	126	126	126	126	126
	Feeding Time [hr]	3	3	3	3	3
WT	Waiting time [hr]	1	1	1	1	1
SF	STY [g]	33.6	37.8	41.6	16.8	50.4
	2-EHA [g]	8.4	4.2	0.42	4.2	12.6
	Feeding Time [hr]	1	1	1	1	1
IF	V-50 [g]	1.8	1.8	1.8	1.5	2
	Water [g]	18	18	18	19.5	20
	Feeding Time [hr]	6	6	6	6	6

Table S2. Reaction formulations for different core-shell particles for samples *f*-*j*. All reactions were run at 80 °C with a stirring speed of 200 rpm.

	Sample	*f*	*g*	*h*	*i*	*j*
IC	MAPTAC (50 wt.%) [g]	15.1	15.1	15.1	15.1	15.1
	Water [g]	370	370	370	370	370
IS	V-50 [g]	0.95	0.9	0.9	0.9	0.9
	Water [g]	10	10	10	10	10
CF	STY [g]	47.2	35	35	35	52.5
	2-EHA [g]	141.8	105	105	105	157.5
	Feeding Time [hr]	3.5	2.5	2.5	2.5	4
WT	Waiting time [hr]	1	1.5	1.5	1.5	-
SF	STY [g]	16.8	56	63	69.3	-
	2-EHA [g]	4.2	14	7	0.7	-
	Feeding Time [hr]	0.5	1.5	1.5	1.5	-
IF	V-50 [g]	1.8	1.8	1.8	1.8	1.8
	Water [g]	18	18	18	18	18
	Feeding Time [hr]	6	6.5	6.5	6.5	6

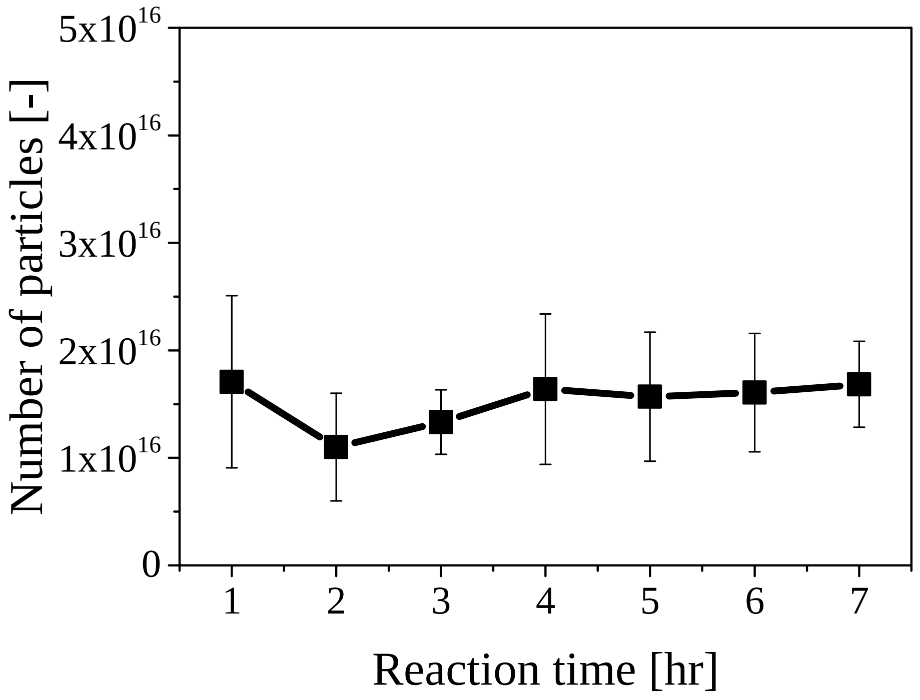

Figure S1. Number of polymer particles in the reactor during the synthesis of sample *a*.

2. Syntheses results

Table S3. Surface zeta potential, pH value and film-forming abilities of synthesized latexes.

Sample	Zeta potential [mV]	pH	Film-forming
a	48.8	6.75	Yes
b	54.5	6.32	Yes
c	51.8	6.69	Yes
d	50.8	6.69	Yes
e	50.9	6.75	Yes
f	50.5	6.46	Yes
g	47.4	6.44	Yes
h	50.8	6.11	Yes
i	50.0	6.52	Yes
j	52.2	6.27	Yes

3. Polymer-cement membranes

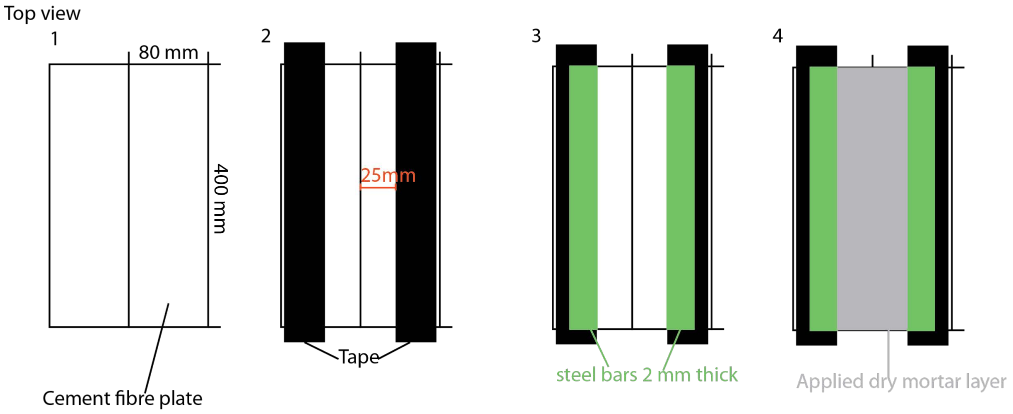

Figure S2. Sketch of the membrane preparation procedure for crack-bridging tests.

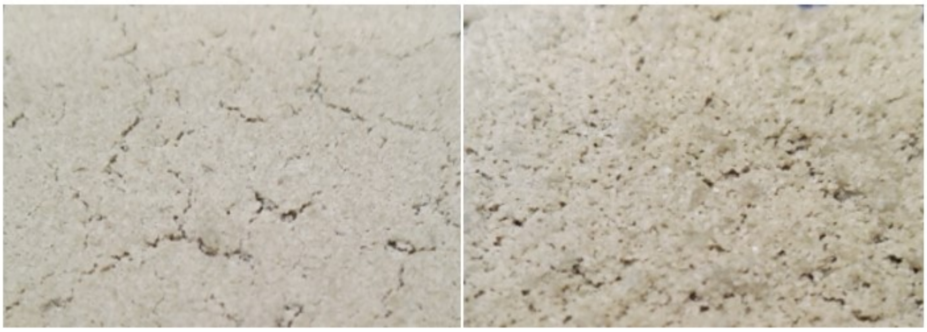

Figure S3. Cement-based membrane obtained with polymer *h* (left) and *i* (right), after spray drying.

4. NMR results and evaluation

The recorded NMR spectra were evaluated by integrating the peaks to measure the polymer composition. The full results are shown in Table S4. The samples taken after one hour had a too low concentration for a reasonable evaluation as the conversion was still relatively low.

Table S4. 2-EHA content of the cumulative polymer particles during the reaction.

Sample	2 hr	3 hr	4 hr	5 hr	6 hr	7 hr
a	0.76	0.73	0.74	0.71	0.69	0.66
b	0.71	0.70	0.73	0.74	0.69	0.66
c	0.73	0.76	0.74	0.75	0.7	0.67
d	0.68	0.68	0.72	0.74	0.73	0.72
e	0.73	0.70	0.71	0.73	0.68	0.65
f	0.69	0.68	0.7	0.73	0.73	0.72
g	0.72	0.71	0.74	0.7	0.63	0.61
h	0.76	0.69	0.72	0.69	0.61	0.59
i	0.77	0.78	0.73	0.69	0.59	0.53
j	0.73	0.77	0.74	0.74	0.76	0.75

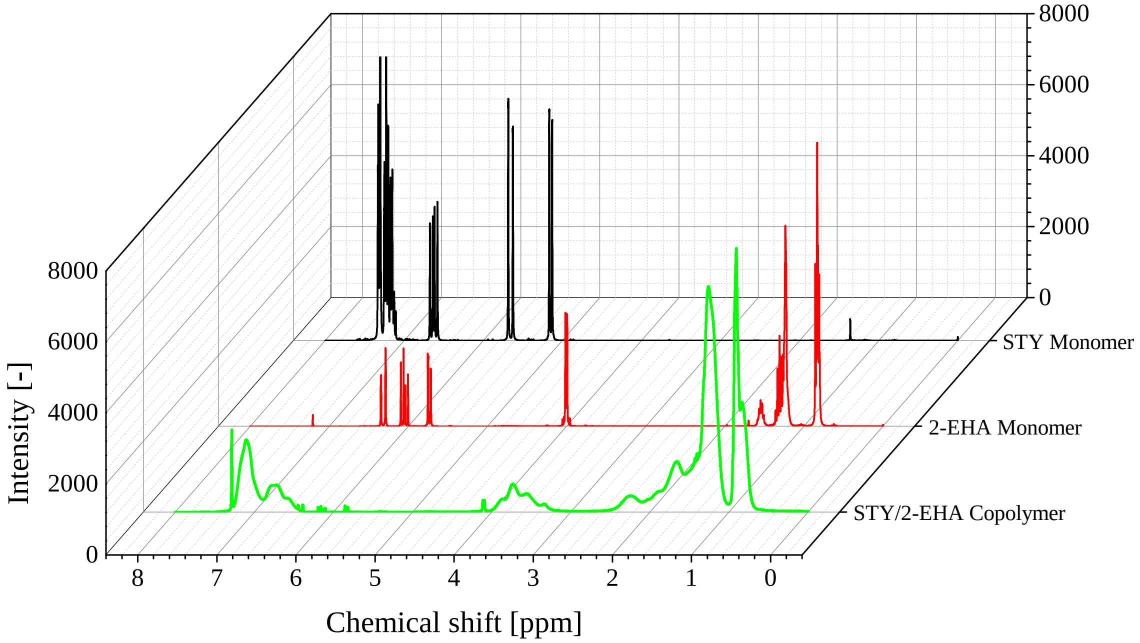

Figure S4. NMR spectra for the monomers STY (black curve) and 2-EHA (red curve) and for the copolymer STY/2-EHA *a* (green curve).
